# Efficacy and Safety of Telbivudine Compared to Entecavir Among HBeAg+ Chronic Hepatitis B Patients: a Meta-Analysis Study

**DOI:** 10.5812/hepatmon.7862

**Published:** 2013-05-23

**Authors:** Jian Liang, Man Jun Jiang, Xin Deng, Xiao Xiao Zhou

**Affiliations:** 1 Department of Hepatology, Ruikang Hospital, Guangxi Traditional Chinese Medical University, Nanning, China

**Keywords:** Hepatitis B, Safety, Meta-Analysis as Topic

## Abstract

**Background:**

Hepatitis B virus (HBV) infection is a serious global health problem that is associated with huge social and economic costs. Early antiviral drugs, such as interferon-α2b, peginterferon-α2a, lamivudine, and adefovir, all have their limitations (such as low responses or safety concerns) in clinical application. Telbivudine and entecavir are two of the latest nucleotide drugs and both have been shown to have potent viral suppression. However, in patients with hepatitis B e antigen (HBeAg)-positive chronic hepatitis B (CHB), inconsistent results have been generated for efficacy between telbivudine and entecavir. Therefore, evidence-based medical data are required to compare the efficacies, in terms of virological and biochemical responses, and safety between telbivudine and entecavir.

**Objectives:**

We aimed to compare the early antiviral efficacy and safety of telbivudine and entecavir in the treatment of patients with hepatitis B e antigen (HBeAg)-positive chronic hepatitis B (CHB).

**Patients and Methods:**

A search for relevant randomized controlled trials (RCTs) on HBeAg-positive CHB patients treated with telbivudine and entecavir for 24 or 52 weeks, published before December 2011, was performed. Primary efficacy endpoint was the cumulative rate of undetectable HBV DNA, and secondary efficacy endpoints included rates of alanine aminotransferase (ALT) normalization, HBeAg disappearance, HBeAg seroconversion and adverse events. Meta-analysis was performed using the Review Manager v5.1.4 software package. We assessed the pooled risk ratios (RRs) and 95% confidence intervals (CIs) using the fixed-or random-effects model.

**Results:**

Six randomized controlled trials (RCTs) involving 555 patients were included. Telbivudine was associated with significantly higher rates of HBeAg disappearance (RR = 1.46, 95% CI: 1.11 - 1.91) and HBeAg seroconversion (RR = 1.76, 95%CI: 1.25-2.48) than entecavir, but had higher adverse events (RR = 2.11, 95%CI: 1.23 - 3.60), compared with entecavir. There was no difference between telbivudine and entecavir in the rate of cumulative undetectable HBV DNA (RR = 0.99, 95% CI: 0.90 - 1.10) and ALT normalization (RR = 0.93, 95% CI: 0.85 - 1.00).

**Conclusions:**

Telbivudine is associated with significantly higher rates of HBeAg disappearance and HBeAg seroconversion than entecavir, whereas entecavir is superior to telbivudine in safety. Both drugs have similar efficacy on rates of cumulative undetectable HBV DNA and ALT normalization.

## 1. Background

Chronic hepatitis B (CHB) is a chronic necrotizing inflammatory disease of the liver caused by persistent hepatitis B virus (HBV) infection, which is defined when a person is positive with hepatitis B surface antigen (HBsAg) for more than 6 months, still with HBsAg and/or is HBV DNA positive (1). It is estimated that approximately 2 billion people are infected with HBV worldwide and 350 million suffer from chronic infection, which results in approximate 500,000 deaths every year, mainly due to its complications including cirrhosis, and hepatocellular carcinoma (2, 3). Therefore, HBV infection is a serious global health problem. For example, over $1 billion is spent for hospitalizations related to HBV infection each year in the United Sates. In addition, there are huge social and economic costs associated with the infection (4). The prevalence of HBV infection is globally uneven and is a significant burden for South Asia, Africa, the South Pacific Islands, the Middle East, the European Mediterranean, The Arctic, South America, Eastern Europe and the Caribbean (1). CHB is a dynamic process, which is influenced by various factors, including viral genotypes, concurrent viral infections, demographic features, and social and environmental factors. At present, vaccination and antiviral therapy are the primary choice for the prevention and treatment of HBV infection. Vaccination can reduce HBV infection effectively in newborn infants of HBsAg-positive mothers (5), but is unsatisfactory for adults. Therefore, effective antiviral therapy is an important intervention for the control of CHB and the progression of complications in adults. Currently, seven agents have been approved by The U.S. Food and Drug Administration (FDA) for the treatment of HBV infection in adults. They are categorized as either interferons (interferon-α2b and peginterferon-α2a) or nucleoside/nucleotide analogues (lamivudine, adefovir, entecavir, telbivudine and tenofovir), which can be used alone or in combination (6). The key point for successful CHB treatment is to apply the standard antiviral therapy. Early antiviral drugs, such as interferon-α2b, peginterferon-α2a, lamivudine, and adefovir, all have their limitations in clinical application. Interferon-α2b has been reported to achieve virological (i.e. the cumulative rate of undetectable HBV DNA) and biochemical (i.e. the rate of normalization of alanine aminotransferase (ALT)) responses of approximately 30% and 23%, respectively (6). Meanwhile, clinical studies have shown that the efficacy of peginterferon-α2a is similar or slightly higher compared with interferon-α2b, and both are associated with many adverse events and expensive costs (1). Lamivudine has a high response rate for the patients who have never received treatment for HBV infection (7); however, long-term use could lead to the development of lamivudine resistance (8). Adefovir is efficacious for lamivudine-resistant HBV (9); however, long-term use may result in kidney impairment and creatine kinase changes (10). Telbivudine and entecavir are two of the latest nucleotide drugs. Telbivudine, a nucleoside analog and an HBV polymerase inhibitor, was approved by the FDA in October 2006, and has potent and specific anti-HBV activity, at the recommended dose of 600 mg/d (11). Telbivudine is safe, effective and well-tolerated (12-16). Entecavir, a new generation of anti-HBV deoxyguanosine nucleoside analog, was approved by the FDA in March 2005 for the treatment of CHB, and the recommended dose is generally 0.5 mg/d or 1 mg/d (17). Clinical studies have indicated that, like telbivudine, this drug is safe and well-tolerated, and is a potent antiviral drug with a low rate of resistance (18-22). Randomized clinical trials have demonstrated that both telbivudine and entecavir have potent viral suppression (23, 24). However, in patients with hepatitis B e antigen (HBeAg)-positive CHB, even though, some studies have shown similar efficacy between telbivudine and entecavir regarding the rates of HBeAg seroconversion, ALT normalization and HBeAg disappearance (25-28), others have failed to support these results (29, 30). Therefore, evidence-based medical data are required to compare the efficacies, in terms of virological and biochemical responses, and safety between telbivudine and entecavir.

## 2. Objectives

The aim of the present Meta-analysis of the related studies published to date in peer-reviewed journals was to examine and compare the early efficacies, and safety between telbivudine and entecavir in the treatment of patients with HBeAg-positive CHB.

## 3. Patients and Methods

### 3.1. Search Strategy

The PubMed, Embase, Cochrane library, China National Knowledge Infrastructure (CNKI), and Wanfang databases were searched for relevant studies published up to December 2011. A highly sensitive search strategy was used to identify randomized controlled trials (RCTs) with a combination of MeSH headings and text words relating to (i) telbivudine, (ii) entecavir, and (iii) chronic hepatitis B, and the synonyms of each word. Initially, the title and abstract identifying relevant studies were screened and examined and this excluded any obviously irrelevant studies. Then, the full-texts of the pertinent articles were retrieved and used to determine the relevancy of the study design and data, according to the inclusion and exclusion criteria detailed below. Additional studies were identified by screening the reference lists of each relevant study. Furthermore, reviews concerning the relevant topic were retrieved from the above-mentioned databases so as to potentially broaden the search by identifying additional relevant publications from the studies cited in the reviews.

### 3.2. Criteria for Study Inclusion and Exclusion

Study design; RCTs, without limitation of language, type of publication and whether or not a blind method was present were included. Study patients; eligible patients were aged from 18 to 65 years and had previously untreated HBeAg-positive CHB, regardless of race, nationality and gender. Diagnostic criteria for CHB included: (i) detectable HBsAg for ≥ 24 weeks prior to screening, (ii) serum HBV DNA levels of ≥ 20,000 IU/ml (105 copies/ml), (iii) persistent or intermittent elevated levels of ALT/aspartate aminotransferase (AST), and (iv) moderate or severe chronic hepatitis as shown by histology. Exclusion criteria included: (i) coinfection with human immunodeficiency virus or other forms of hepatitis virus, (ii) combined liver cirrhosis, or hepatocellular carcinoma, and (iii) the use of other antiviral drugs at the same time. In addition, if two or more studies were based on the same or had overlapping subjects, only the study referring to the largest number of subjects was selected for inclusion in the Meta-analysis. Interventions; the telbivudine group received 600 mg/day while the entecavir group received 0.5 mg/day.

### 3.3. Data Extraction and Quality Assessment

After the eligible studies had been identified, two independent investigators performed the data extraction from the studies. A third investigator resolved inconsistencies, and a consensus was achieved for all data prior to the Meta-analysis. The following information was collected from each publication: study characteristics (such as the first author’s name, publication year, and number of patients), interventions, and endpoint assessments. Methodological quality assessment of the included RCTs was performed using the domains (randomization, allocation concealment, blinding, complete outcome data, selective outcome reporting, and other potential biases) described by Higgins et al. (31) in the Cochrane Reviewers’ Handbook 5.1.4.

### 3.4. Assessments of Endpoints

Primary efficacy endpoint: the cumulative rate of undetectable HBV DNA. Secondary efficacy endpoints: rates of ALT normalization, HBeAg disappearance, HBeAg seroconversion and adverse events.

### 3.5. Statistical Analysis

To evaluate early antiviral efficacy and safety of telbivudine and entecavir in the treatment of patients with HBeAg-positive CHB, risk ratios (RRs) with 95% confidence intervals (CIs) were calculated using pooled group and subgroup data from the studies. Data pooling was carried out by using the fixed effects model (based on the Mantel-Haenszel method) or the random effects model (based on the Dersimonian and Laird method) (32, 33). The random effects model was used if heterogeneity existed between the studies from which the data was extracted; if not, the fixed effects model was used. Heterogeneity among studies was assessed by the Chi-square-based Q test and Ι2, and heterogeneity was considered significant when the two-tailed P value was less than 0.1. Ι2 was used to quantify variation in RR that was attributable to heterogeneity. Publication bias was estimated by using the Begg’s test and the Egger’s test (34, 35). Finally, the statistical significance of the RR was determined by using the Z test. Subgroup analyses were performed for different endpoints and for treatment durations. All P values were calculated using a two-tailed analysis. For all tests, a P value of < 0.05 was considered statistically significant, except for heterogeneity. All statistical analyses were performed with the Review Manager v5.1.4 software package (http://ims.cochrane.org/revman).

## 4. Results

### 4.1. Search Results

**Figure 1.**
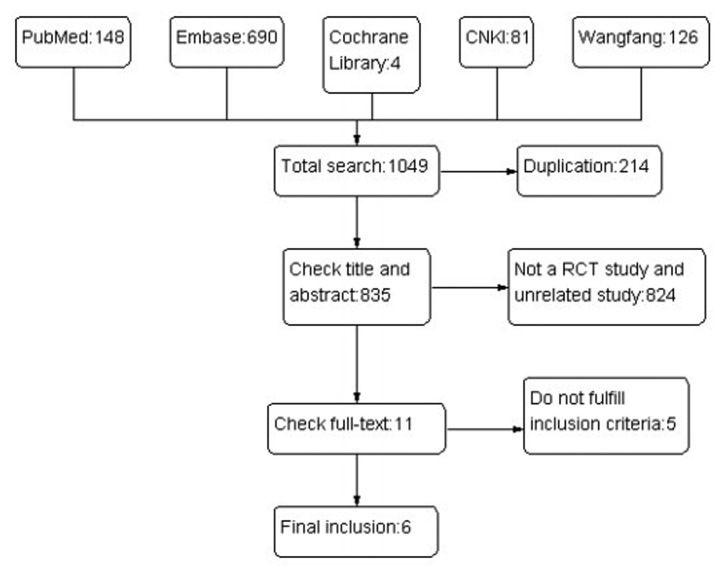
The Flowchart of the Literature Search

The results of the literature search are summarized in Figure 1.

A total of 1049 studies were identified, and the titles and abstracts were reviewed for initial screening based on the described search strategies. Eleven studies were chosen for a detailed review, and six studies that met the inclusion and exclusion criteria were included in the Meta analysis (25 - 30), with a total of 555 CHB patients, 277 in the telbivudine group and 278 in the entecavir group. The characteristics of each study are summarized in Table 1.

**Table 1.** The Characteristicsof Studies Included in the Meta Analysis

Study	Patient Number		Intervention, mg qd		Gender,No.		Age,Mean ± SD, y		Duration, wk^a^	End point^b^
	LdT^a^	ETV	LdT	ETV	Male	Female	LdT	ETV^a^		
**Huang et al. 2011** **,** **(** **23** **)**	90	90	600	0.5	123	57	28.8+9.8	31.0+1.0	52	1, 2, 3, 4
**Shi et al. 2008,** **(** **24** **)**	40	40	600	0.5	54	26	30.5+7.1	31.5+7.95	24	1, 2, 3, 4, 5
**Suh** ** et al. 2010** **,** **(** **29** **)**	23	21	600	0.5	30	14	36.2+9.6	33.4+8.82	12	1, 5
**Xu** ** et al. 2011** **,** **(** **26** **)**	30	30	600	0.5	41	19	32.7+10.6	33.6+8.8	24	1, 2, 3, 4, 5
**Zheng** ** et al. 2010** **,** **(** **27** **)**	65	66	600	0.5	91	40	31.6+8.7	33.5+9.1	24	1, 2, 3, 4, 5
**Zhu et al. 2011** **,** **(** **30** **)**	30	30	600	0.5	55	5	28.0+9.1	31.8+7.1	24	1, 2, 3, 4, 5

^a^Abbreviations: ETV, entecavir; LdT, telbivudine; wk, week

^b^1, The cumulative rate of undetectable HBV DNA; 2, ALT normalization rate; 3, HBeAg disappearance rate; 4, HBeAg seroconversion rate; 5, adverse event incidence

### 4.2. Methodological Quality Assessment of the Included Studies

The methodological quality of each study is summarized in Table 2. The quality was high for two, moderate for one and low for three studies.

**Table 2.** Assessment of Methodological Quality of the Included Studies

Studies Included	Randomization	Allocation Concealment	Blinding	Complete Outcome Data	Selective Outcome Reporting	Other Potential Sources of Bias
**Huang et ** **al.** ** 2011** **(** **23** **)**	Low risk	Unclear	Unclear	Unclear	Low risk	Unclear
**Shi et al.** ** 2008** **(** **24** **)**	Low risk	Unclear	Unclear	Unclear	Low risk	Unclear
**Suh** ** et al. ** **2010** **(** **29** **)**	Low risk	Unclear	Low risk	low risk	Low risk	Unclear
**Xu** ** et al** **.** ** 2011** **(** **26** **)**	Low risk	Unclear	Unclear	Unclear	Low risk	Unclear
**Zheng** ** et al** **.** ** 2010** **(** **27** **)**	Low risk	Low risk	Low risk	Low risk	Low risk	Unclear
**Zhu et al.** ** 2011** **(** **30** **)**	Low risk	Low risk	Unclear	Unclear	Low risk	Unclear

### 4.3. Meta-Analysis of the Cumulative Rate of Undetectable HBV DNA 

**Figure 2.**
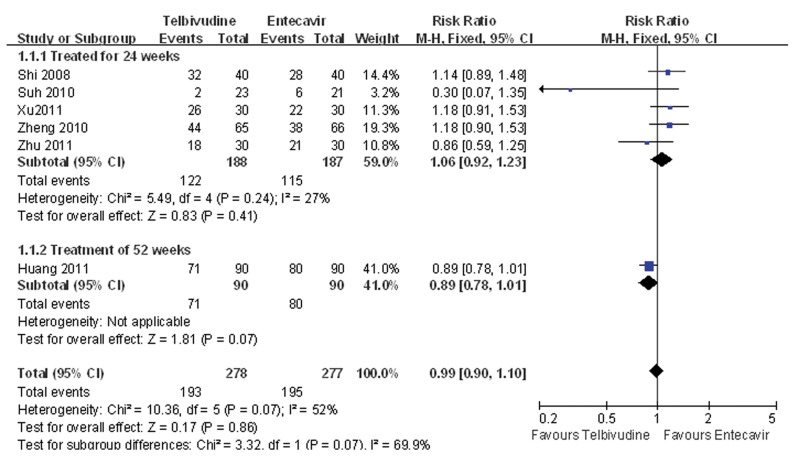
Comparison of the Cumulative Rate of Undetectable HBV DNA Between Telbivudine and Entecavir in the Treatment of Patients With HBeAg-Positive Chronic Hepatitis B

Five of the six studies compared the cumulative rate of undetectable HBV DNA at 24 weeks after treatment between the telbivudine and entecavir groups (25-28, 30). The P value was 0.24 for the heterogeneity, and the corresponding I2 statistic was 27%, suggesting low variability among the studies. However, the P values were P = 0.22 and P = 0.03 for the Begg’s and the Egger’s tests, respectively, indicating a high probability of publication bias. The Meta-analysis showed that the cumulative rates of undetectable HBV DNA were similar between the two groups (fixed-effects model: RR = 1.06, 95% CI: 0.92-1.23, P = 0.41; Figure 2). Only one study compared the cumulative rate of undetectable HBV DNA at 52 weeks between the telbivudine and entecavir groups, and demonstrated no significant difference (RR = 0.89, 95% CI: 0.78-1.01, P = 0.07; Figure 2) (29).

### 4.4. Meta Analysis of ALT Normalization Rate

Five of the six studies compared the ALT normalization rate between the telbivudine and entecavir groups (25, 26, 28-30). The P value was 0.24 for heterogeneity, and the corresponding I2 statistic was 27%, suggesting a low variability among the studies.

**Figure 3.**
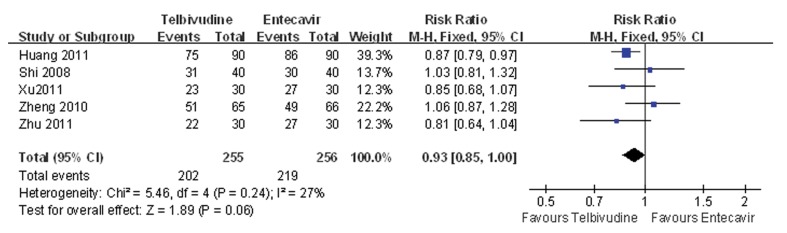
Comparison of the ALT Normalization Rate Between Telbivudine and Entecavir in the Treatment of Patients With HBeAg-Positive Chronic Hepatitis B

The P values were P = 1.00 and P = 0.62 for the Begg’s and the Egger’s tests, respectively, indicating a low probability of publication bias. The Meta-analysis showed that there was no difference between the telbivudine and entecavir groups in ALT normalization rate (fixed-effects model: RR = 0.93, 95%CI: 0.85-1.00, P = 0.06; Figure 3).

### 4.5. Meta-analysis of HBeAg Disappearance Rate

**Figure 4.**
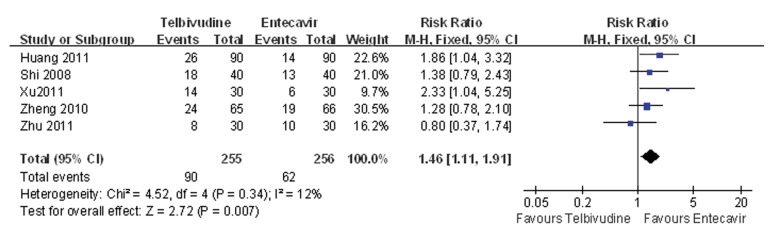
Comparison of HBeAg Disappearance Rate Between Telbivudine and Entecavir in the Treatment of Patients With HBeAg-Positive Chronic Hepatitis B

Five of the six studies compared the HBeAg disappearance rate between the telbivudine and entecavir groups (25, 26, 28-30). The P value was 0.34 for the heterogeneity, and the corresponding I2 statistic was 12%, suggesting low variability among the studies. The P values were P = 0.46 and P = 0.94 for the Begg’s and the Egger’s tests, respectively, indicating a low probability of publication bias. The Meta-analysis showed that the telbivudine group had a significantly higher rate of HBeAg disappearance than the entecavir group (fixed-effects model: RR = 1.46, 95%CI: 1.11 -1.91, P = 0.007; Figure 4).

### 4.6. Meta-analysis of HBeAg Seroconversion Rate

**Figure 5.**
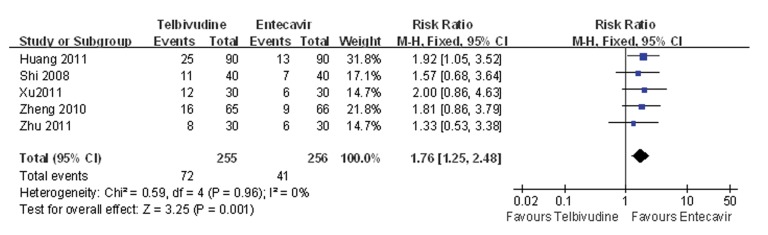
Comparison of the HBeAg Seroconversion Rate Between Telbivudine and Entecavir in the Treatment of Patients With HBeAg Positive Chronic Hepatitis B

Five of the six studies compared the HBeAg seroconversion rate between the telbivudine and entecavir groups (25, 26, 28-30). The P value was 0.96 for the heterogeneity, and the corresponding I2 statistic was 0%, suggesting low variability between studies. The P values were P = 0.09 and P = 0.21 for the Begg’s and the Egger’s tests, respectively, indicating a low probability of publication bias. The Meta-analysis showed that the telbivudine group was associated with a significantly higher rate of HBeAg seroconversion than the entecavir group (fixed-effects model: RR = 1.76, 95%CI: 1.25-2.48, P = 0.001; Figure 5).

### 4.7. Meta-analysis of Adverse Events Rate

**Figure 6.**
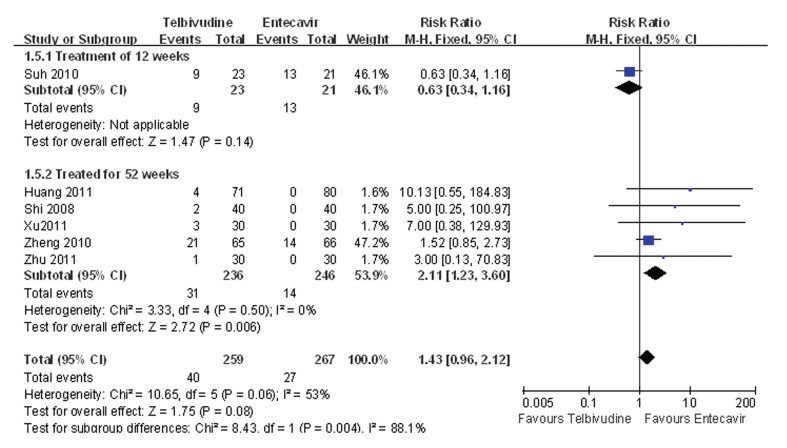
Comparison of the Adverse Event Incidence Between Telbivudine and Entecavir in the Treatment of Patients With HBeAg-Positive Chronic Hepatitis

One study compared the adverse event incidence during the 12-week treatment between the telbivudine and entecavir groups, and observed that the difference was not statistically significant (RR = 0.63, 95%CI: 0.34-1.16, P = 0.14; Figure 6) (27). Five studies reported incidence of an adverse event during the 52-week treatment (25, 26, 28-30), and the P value was 0.50 for the heterogeneity, and the corresponding I2 statistic was 0%, suggesting low variability among the studies. The P values were P = 1.00 and P = 0.06 for the Begg’s and the Egger’s tests, respectively, indicating a low probability of publication bias. The Meta-analysis showed that the entecavir group was superior to the telbivudine group in terms of adverse event incidence (fixed-effects model: RR = 2.11, 95%CI: 1.23-3.60, P = 0.006; Figure 6).

## 5. Discussion

In the present study, the Meta-analysis showed that telbivudine was associated with significantly higher rates of HBeAg disappearance and HBeAg seroconversion compared with entecavir, but entecavir was superior to telbivudine in safety profiles. There was no difference between telbivudine and entecavir in rates of cumulative undetectable HBV DNA and ALT normalization. This Meta-analysis included six RCTs and all of them described specific randomization methods. Two RCTs used appropriate allocation concealments whereas the remaining four did not describe whether or not allocation concealment was used and therefore were considered as having a low risk of selection bias. The use of blinding was reported by two RCTs, but this remained unknown for the other four studies, resulting in a moderate risk of measurement bias. Two RCTs reported losses to follow-up, drop-outs and used intention-to-treat analysis; in contrast, the other four RCTs did not describe incomplete outcome data, and therefore, there was a moderate risk of follow-up bias. None of the four RCTs selectively reported outcomes, so the risk of reporting bias was low. When a Meta-analysis based on published literature was conducted, the publication bias could not be ignored. The evidence of bias existence in favor of publication of statistically significant results is well-documented (36-38). Nevertheless, the possibility of important selection or publication bias of our results is small as a low probability of publication bias was observed in the Meta-analysis for ALT normalization rate, HBeAg disappearance rate, HBeAg seroconversion rate and adverse events rate. The results of our Meta-analysis indicate that, on one hand, telbivudine displays distinct advantages in HBeAg disappearance and HBeAg seroconversion. On the other hand, it has been reported that entecavir is associated with a lower incidence of adverse events, which is related to its potent anti-HBV effect and genetic barrier of resistance (39). However, none of the six included studies evaluated the long-term outcomes such as hepatocellular carcinoma and mortality in patients with HBeAg positive CHB treated with telbivudine and entecavir. Therefore, more RCTs, especially those with multi-centers and large samples assessing the long-term consequences are required. According to the findings of the present study and those from previous studies, telbivudine is more suitable for CHB patients with a high HBeAg level, owing to its high rates of HBeAg disappearance and HBeAg seroconversion. Entecavir is more suitable for CHB patients with active HBV DNA duplication and resistance (40), owing to its low incidence of adverse events. There are several limitations in the present study. Firstly, we did not perform a literature search for unpublished relevant studies, or for the original data of the included studies. However, since we did not impose any limitations for language, place of publication and quality, we believe that the literature search was sufficient, which can be reflected by the number of studies found initially (n = 1049). Secondly, there were different treatment durations in the included RCTs, and duration of 24 weeks was the most common. We conducted subgroup analyses according to the duration of treatment to minimize the impact of treatment duration on the conclusions. Thirdly, the included RCTs were all conducted by large tertiary teaching hospitals; thus, there may be selection bias in the choice of the study population and the findings in the present study may be more applicable to the patients of large tertiary teaching hospitals. In conclusion, telbivudine is associated with significantly higher rates of HBeAg disappearance and HBeAg seroconversion than entecavir, whereas entecavir is superior to telbivudine in safety. Both drugs have similar efficacy in terms of rates of cumulative undetectable HBV DNA and ALT normalization. Further studies, particularly RCTs with multi-centers and large samples assessing the long-term consequences are required to confirm the efficacy and safety of telbivudine and entecavir in the treatment of patients with HBeAg-positive CHB.
